# Examining profiles of convergence and divergence in reports of parental warmth: Links to adolescent developmental problems

**DOI:** 10.1017/S0954579424000762

**Published:** 2024-04-15

**Authors:** Carlie J. Sloan, Emily Forrester, Stephanie Lanza, Mark E. Feinberg, Gregory M. Fosco

**Affiliations:** 1 Human Development and Family Studies, The Pennsylvania State University, University Park, PA, USA; 2 Edna Bennett Pierce Prevention Research Center, University Park, PA, USA; 3 Mathematica Policy Research Inc, Princeton, NJ, USA; 4 Biobehavioral Health, The Pennsylvania State University, University Park, PA, USA

**Keywords:** Adolescent, informant discrepancies, latent profiles, parent warmth

## Abstract

Parental warmth during the transition from childhood to adolescence is a key protective factor against a host of adolescent problems, including substance use, maladjustment, and diminished well-being. Moreover, adolescents and parents often disagree in their perceptions of parenting quality, and these discrepancies may confer risk for problem outcomes. The current study applies latent profile analysis to a sample of 687 mother–father–6th grade adolescent triads to identify patterns of adolescent–parent convergence and divergence in perceptions of parental warmth. Five profiles were identified, and associations with adolescent positive well-being, substance use, and maladjustment outcomes in 9th grade were assessed. Patterns of divergence in which adolescents had a pronounced negative perception of parental warmth compared to parents, as well as those wherein pronounced divergence was present in only one adolescent–parent dyad, were associated with diminished positive well-being compared to adolescents who had more positive perceptions of warmth than parents. Having more negative perceptions of warmth compared to parents was also associated with elevated risk for alcohol and marijuana initiation, but only when the divergence was pronounced rather than more moderate. These findings add nuance to findings from previous between-family investigations of informant discrepancies, calling for further family-centered methods for investigating multiple perspectives.

## Introduction

The developmental changes that occur during adolescence implicate the entire family system. Positive family environmental factors early in adolescence, such as high-quality parenting, can serve a protective function into later years. Examining long-term effects of parenting on salient later adolescent outcomes, like adjustment, well-being, and substance use, is an important line of inquiry. However, parents and adolescents do not often see eye to eye in perceptions of parenting quality; divergent perspectives of parenting are common, especially in early adolescence. Therefore, the potential additional risk or protection conferred by divergent adolescent–parent perceptions of parenting should be explored. Person-centered methodologies represent one means for exploring such associations that can account for the unique contributions of parent and adolescent perspectives simultaneously. The current study reflects a person-centered approach for exploring potential subgroups of families characterized by unique patterns of adolescent–parent convergence and divergence in reports of parenting, and whether these patterns are differentially associated with later adolescent developmental outcomes.

### Capturing parenting skills and adolescent developmental outcomes during key periods

Adolescence is a time of rapid development that often drives reorganization of the family system. Despite adolescents’ growing autonomy, a warm adolescent–parent relationship remains a robust protective factor for a wide range of adolescent developmental outcomes, such as positive well-being and reduced risk for substance use and maladjustment. Adolescents’ anxiety and depressive symptoms, aggression, diminished positive well-being, and early substance use initiation have each been associated with more serious problems in adulthood (Chassin et al., 2004; Johnson et al., 2018; Liu et al., 2013). For instance, early engagement in substance use is associated with increased risk for later substance misuse, as well as other mental and physical health problems like suicidal ideation (Ahuja et al., 2021; Chassin et al., 2004; Sartor et al., 2007). Adolescents with greater mental health problems like anxious/depressive symptoms are more likely to develop major depression and anxiety disorder in adulthood and are also at increased risk of adult substance abuse and dependence (Johnson et al., 2018; McLeod et al., 2016). Similarly, problem behaviors like aggression in adolescence are consistently linked with increased risk for mental health problems and suicidal behavior, as well as greater risk of violent behavior in adulthood, including intimate partner abuse, child abuse, and homicide (Liu et al., 2013). In contrast, those with higher positive well-being in adolescence are better protected from future emotional problems, are more likely to have high work satisfaction, and exhibit better quality social relationships (Richards & Huppert, 2011). Due to the significant threats posed by poor adolescent mental health, substance use, problem behavior, and diminished well-being, understanding the contexts in which these problems arise in adolescence is a key public health concern.

Certain windows of the adolescent developmental period have emerged as key times for assessing such developmental outcomes. The transition to high school may be a particularly salient time to examine adolescents’ adjustment, well-being, and substance use. School transitions represent a time when adolescents are at increased risk for negative adjustment to school as well as poorer adjustment and well-being in other domains (Benner, 2011; Steinberg & Silk, 2002; Akos & Galassi, 2004). The high school transition coincides with new peer contexts, role demands, and behavioral expectations (Benner, 2011), which can increase stressors and threaten adolescents’ well-being and adjustment as well as put them at risk for problem behaviors like substance use (Barber & Olsen, 2004; Benner & Graham, 2009; Newman et al., 2007). Adolescents who engage in substance use before the high school years have also been shown to be at significantly elevated risk for developing substance dependence as adults, among other serious health issues (Grant & Dawson, 1997; Odgers et al., 2008; Sartor et al., 2007), making this a particularly salient time to identify problematic substance use behaviors. Qualities of the family environment, like supportive parenting, can buffer the negative effects of the high school transition on adolescent well-being (Isakson & Jarvis, 1999; Newman et al., 2007; Roderick, 2003). This suggests that the family contextual antecedents of later adolescent risky behaviors, maladjustment, and diminished well-being may be best measured earlier in adolescence.

Adolescents’ transition into middle school represents a developmental milestone during which family relationships and parenting have a pronounced and lasting impact. As adolescents transition to middle school, they experience significant role and responsibility changes, academic challenges, and changes to their social environment that threaten well-being and adjustment (Akos & Galassi, 2004; Barber & Olsen, 2004; Véronneau & Dishion, 2010). High-quality parenting and family relationships can serve a protective role for adolescents at this time, with factors like parent–child attachment and autonomy support reducing the impact of this transition on adolescents’ school adjustment as well as social competence and individual well-being (Bouffard et al., 2023; Duchesne et al., 2009; Lochman & Wells, 2002; Véronneau & Dishion, 2010). Indeed, many of the most promising family-based intervention programs target the family relationships and parenting skills among families of early middle schoolers (Dishion et al., 2002; Patterson et al., 2010; Spoth et al., 1999, 2007; Thomas & Zimmer-Gembeck, 2007), suggesting this as a time when supporting parent skills can have a notable impact. Therefore, capturing measurements of parenting during this early adolescent stage is a useful means of understanding risk for later adolescent behavioral, adjustment, and well-being outcomes.

Evidence points to parental warmth as a particularly critical aspect of parenting for early adolescents. Parental warmth, characterized by behaviors like praise, affection, and nurturance, supports early adolescents’ health and well-being both concurrently and across the adolescent developmental period. Higher parental warmth is associated with reduced risk for adolescent internalizing and externalizing problems (Aquilino & Supple, 2001; Liu et al., 2013; Pinquart, 2017; Rothenberg et al., 2020), across a range of familial and sociocultural factors; inconsistent warmth also places adolescents at risk for externalizing problems (Zheng & McMahon, 2022). Warm parenting is also positively associated with adolescent’s life satisfaction and happiness (Bülow et al., 2022; Operario et al., 2006). In other work, parenting styles characterized by low warmth have been associated with adolescents’ elevated risk of drug and alcohol use (Johnson & Pandina, 1991; Montgomery et al., 2008). Adolescents who perceive their parents as warm and supportive tend to disclose more information about their activities, whereabouts, and peer relationships (Branje, 2018) – a critical factor for parents’ ability to monitor adolescent activities and prevent delinquency and substance use engagement (Keijsers, 2016; Stattin & Kerr, 2000). Although declines in parental warmth across adolescence are normative (Shanahan et al., 2007; Steinberg & Morris, 2001), families in which supportive parenting is high in early adolescence are better protected from such declines (Laursen et al., 2010). Warm and supportive parenting early in adolescence has therefore been identified as one key protective factor against negative developmental outcomes.

In addition to parenting quality itself, adolescent impressions of parenting may play an additional role in identifying early adolescent risk for later developmental problems. The normative biological and cognitive transitions that occur in early adolescence prompt a process by which the adolescent–parent relationship reorganizes to become more egalitarian and reciprocal in response to adolescents’ growing capability for autonomy (Branje, 2018; Steinberg & Morris, 2001). Alongside this role adjustment, parents’ beliefs about their child’s changing developmental needs may lead them to be less supportive and involved in their early adolescents’ lives (Dishion et al., 2004). Parents may perceive their reductions in involvement with their early adolescents’ lives as scaffolding their child’s growth; however, parent warmth in the early adolescent years may still serve a critical role in protecting against negative distal outcomes. For some adolescents, parents’ reductions in involvement early in adolescence may be perceived as a lack of parent support. This may result in divergent perspectives of parenting quality by parents and adolescents. Indeed, researchers using multi-informant methods for studying family relationships and functioning find only small to medium correlations between parent and adolescent reports of parenting behaviors (Gaylord et al., 2003; Guion et al., 2009; Hou et al., 2020; Van Heel et al., 2019; Vrolijk et al., 2023). Additionally, meta-analyses and longitudinal studies have found that adolescent–parent divergence may be particularly pronounced earlier in adolescence compared to later (Gniewosz et al., 2023; Hou et al., 2020; Mastrotheodoros et al., 2018). Therefore, early adolescence may represent a particularly meaningful time for assessing divergent family perspectives. The implications that early adolescent–parent divergence in perspectives of the family have for adolescent development has since been studied using a variety of theoretical and methodological approaches.

### Making meaning of adolescent–parent discrepancies

Convergence and divergence in parents’ and adolescents’ perspectives of the family are theorized to have important developmental implications, according to the Operations Triad Model (De Los Reyes et al., 2013). *Convergence* is indicated when both informants’ reports lead to the same conclusion (e.g., parental warmth in a given family is high, as indicated by either the parent report or the adolescent report). *Divergence* is indicated when informant reports are different to a meaningful extent (e.g., parental warmth is high according to parent reports, but moderate or low according to adolescent reports). In some cases, divergence across informants may be explained by issues of measurement reliability or validity (De Los Reyes et al., 2013). However, considerable research has found that both adolescents and parents provide reliable and valid reports of the family, wherein informant discrepancies reflect relevant information for understanding the construct at hand (De Los Reyes et al., 2023a, 2023b). Therefore, adolescent–parent informant discrepancies are thought to reflect meaningful variation in parent and adolescent perceptions of the family. Understanding the implications of informant convergence and divergence for adolescent development are best understood through careful consideration of the nature of the constructs and the degree of divergence observed.

### Convergence and divergence: risk, protection, and magnitude

When conceptualizing risk with multi-informant family data, there are several qualities that should be considered. When convergent reports are present, risk and protection conferred can be understood based on whether the convergence is on a risk factor or protective factor. As De Los Reyes and Ohannessian (2016) note, adolescent–parent convergence on reports of protective factors, such as high parental monitoring or parental acceptance, is related to lower risk for maladjustment. For example, adolescent–parent convergence on high reports of relationship quality, parental monitoring, and parental acceptance has been associated with lower levels of maladjustment and substance use risk (Gniewosz et al., 2023; Laird & De Los Reyes, 2013; Lippold et al., 2014; Nelemans et al., 2016). In contrast, convergence on high levels of risk factors may be a strong indicator (even stronger than divergent reports) of maladjustment risk, as it may indicate that the risk factor has been pervasive in the family for a long period of time and across adolescent–parent interactions (De Los Reyes & Ohannessian, 2016). To offer empirical examples: recent studies document relations in which adolescent–parent convergence in reports of poor adolescent–parent relationship quality and high parental control were associated with the adolescent depressive symptoms and externalizing behaviors, respectively (Nelemans et al., 2016; Van Heel et al., 2019). These studies demonstrate that adolescent–parent convergence on high levels of risk or protective factors can be a meaningful indicator of adolescent adjustment and well-being trajectories.

The magnitude and direction of the divergence in parent and adolescent reports is also thought to provide meaningful information about adolescent risk outcomes. In one sense, moderate divergence in adolescent–parent reports of family functioning may be indicative of a natural and healthy process in which developing adolescents are individuating away from the family and seeking more autonomy (Allison & Sabatelli, 1988; Bowen, 1978). However, divergent perspectives on family functioning that are larger in magnitude are likely indicative of maladaptive processes that pose a risk for adolescent development. The majority of the evidence for informant discrepancies points to divergent reports as signaling deeper processes of poor communication, lack of parental awareness, or family disagreement, which undermine adolescent adjustment and positive well-being (De Los Reyes et al., 2019; De Los Reyes & Ohannessian, 2016).

Specific patterns of adolescent–parent divergence in perspectives seem to pose particular risk for healthy adolescent development. Across studies, adolescents typically report poorer perceptions of family functioning than their parents (Hou et al., 2020). This pattern of *negative adolescent*–*parent divergence* (i.e., a discrepancy in which adolescent scores are more negative in valence than parent scores) is associated with psychological maladjustment. For example, adolescents’ poorer perceptions of parental warmth compared to parents has been associated with diminished daily mood (Janssen et al., 2023) as well as longer-term adjustment problems (Gniewosz et al., 2023; Vrolijk et al., 2023). Similarly, adolescents who perceived their mothers were more overprotective compared to mothers’ self-reports were at elevated risk for externalizing problems (Van Petegem et al., 2020). Divergent adolescent–parent perceptions of parental discipline and nurturance have also been associated with adolescents’ later internalizing problems (Guion et al., 2009). Taken together, this body of work implies that negative adolescent–parent divergence in reports of parenting often signals family environments that lack warmth and strong adolescent–parent bonds, posing a risk to adolescent well-being and adjustment across multiple domains.

Because the majority of research has documented that adolescents typically report poorer family functioning compared to parents, this pattern of negative adolescent–parent divergence has dominated the focus of research studies to date. However, these studies have mostly relied on variable-centered methods, potentially overlooking the possibility that a subset of families exhibit a *positive adolescent*–*parent divergence* (i.e., adolescents report more favorable family functioning than parents). Studies using advanced statistical approaches like latent profile analysis and latent congruence modeling have documented a pattern in which adolescents’ reports of parenting positively diverge from parents and that this may be protective against later maladjustment (De Los Reyes et al., 2010; Gniewosz et al., 2023; Hou et al., 2018; Mastrotheodoros et al., 2018; Rote & Smetana, 2016). In a study of daily family relationships, Brinberg et al. (2017) found that a meaningful proportion of families (about 15%) could be characterized by positive adolescent–parent divergence at the daily level, and that this pattern was protective against behavior problems and depression compared to other patterns of adolescent–parent divergence and convergence. Therefore, positive adolescent–parent divergence may represent a healthy pattern of family functioning similar to adolescent–parent convergence on protective factors. To gain more insights into the diverse patterns of family relationships, it is essential to leverage statistical approaches that incorporate the full breadth of patterns of adolescent–parent divergence.

### Accounting for the unique contributions of multiple parents

For adolescents who have multiple parental figures who attend to their needs, such as those living in two-parent households, it is likely that parents have both overlapping as well as unique contributions to parenting, which may in turn have distinct implications for adolescent development. Historically, mothers and fathers have been socialized to contribute to the family system in different ways (Cabrera et al., 2014; Palkovitz et al., 2014). Because of this difference in expected roles, mothers’ and fathers’ shared family values and norms often manifest in different ways behaviorally. Therefore, it is expected that measures of parenting that are rooted in parents’ behavior, such as warmth, will yield different results for mothers and fathers (Palkovitz et al., 2014). The distinct contributions of mother and father warmth for adolescent development have emerged in empirical studies. For example, mothers have exhibited higher scores on measures of warm parenting than fathers (Mastrotheodoros et al., 2018). Warm and supportive parenting from fathers and mothers has been differentially associated with adolescents’ later adjustment (Padilla-Walker et al., 2016), with many studies documenting a stronger relation between father’s parenting and both general and short-term indices of adolescent functioning compared to mothers’ parenting (Gniewosz et al., 2023; Janssen et al., 2023; Larson & Richards, 1994; Padilla-Walker et al., 2016). However, a paucity of research examining the joint influence of mothering and fathering limits our understanding of this relationship.

Patterns of divergence in adolescent–mother and adolescent–father perspectives of parenting may also contribute uniquely to adolescent development, above and beyond levels of parenting quality. Recent studies incorporating both mothers and fathers have helped to elucidate these pathways. Some work suggests that adolescents’ perceptions of parenting may diverge more from mothers’ compared to fathers’ perceptions (Gniewosz et al., 2023; Vrolijk et al., 2023). Studies have also found that adolescents who report poorer parenting than fathers are at the greatest risk for developing adjustment problems, whereas adolescent–mother convergence on poorer reports of parenting posed the most risk for adjustment problems (Nelemans et al., 2016; Vrolijk et al., 2023). On the other hand, many similar patterns of association between adolescent–mother and adolescent–father convergence or divergence and adolescents’ developmental outcomes have also been documented (Gniewosz et al., 2023; Vrolijk et al., 2023). Therefore, methods that can account for the unique impact of mother and father parental warmth while taking a whole-family perspective are needed.

### Examining mother, father, and adolescent perceptions in tandem

Although mother and father warmth may contribute differently to adolescent development, family systems principles urge that adolescent–parent dyads are part of an interdependent and inherently interconnected family system. The systems principal of family *wholism* posits that the subsystems that make up a family, such as parent–adolescent dyads, cannot be understood in isolation but rather in relation to the other family subsystems (Minuchin, 1985). It is widely recognized that fathers’ and mothers’ parenting behaviors, although potentially distinct, are complementary and reciprocally influential (Cabrera et al., 2007). The function of warm and supportive parenting from one parent may therefore differ in the context of relatively higher or lower quality parenting from the other. The interrelatedness of mothering and fathering also has implications for the study of adolescent–parent informant discrepancies. That is, the implications of divergence in one parent–child dyad may differ as a function of the degree of divergence in the other parent–child dyad. However, most studies of adolescent–parent informant discrepancies have only examined one dyad or have examined adolescent–parent dyads separately, leaving a gap in our understanding. Therefore, methodologies that can incorporate adolescents’ parenting experiences across multiple dyads should be employed.

### Toward a holistic conceptualization of family informant discrepancies

Person-centered methods, such as latent profile analysis (LPA; Lazarsfeld & Henry, 1968), offer a holistic evaluation of mother, father, and adolescent perceptions of parenting in multiple ways. First, LPA can capture the potentially unique contributions of mother and father warmth, which can vary in their level, divergence from adolescent perceptions, and implications for adolescent development. Additionally, LPA accounts for each family member’s perspective – as well as their divergence – simultaneously, making LPA particularly suited for capturing the interrelated parent–adolescent relationships within families. In other words, person-centered approaches can move toward conceptualizations of family functioning that are more inclusive of all family member perspectives, while also retaining the ability to parse out the unique contributions of mother’s and father’s parenting quality separately.

There may also be important, non-linear considerations when understanding the implications of adolescent–parent discrepancies (De Los Reyes & Ohannessian, 2016). Specifically, extreme discrepancies in either direction (positive or negative adolescent–parent discrepancies) may be more impactful on adolescent development than moderate discrepancies (e.g., Feinberg et al., 2000). However, traditional methods like linear regression assume that difference scores exist on a linear continuum where change at any given part of the scale is equally meaningful. LPA can advance our knowledge about the implications of more and less extreme patterns of divergence, by identifying the meaningful groupings of families characterized by higher and lower adolescent–parent divergence.

A handful of person-centered studies of adolescent–parent informant discrepancies have been conducted and have supported the idea that negative adolescent–parent divergence, in which adolescents underestimate parenting quality compared to parents, is a risk factor for adolescent problem behaviors and maladjustment problems (De Los Reyes et al., 2010; Hou et al., 2018). These studies have also added information beyond that of variable-centered methods. For instance, subgroups of positive adolescent–parent divergence wherein adolescents report better parenting quality than parents have been observed (De Los Reyes et al., 2010; Hou et al., 2018; Rote & Smetana, 2016), and have been associated with more positive adolescent outcomes (Hou et al., 2018). However, these studies have all been limited to adolescent–mother dyads alone or have examined adolescent–mother and adolescent–father dyads separately, which does not fully represent the adolescents’ lived experience wherein the adolescent–mother relationship is influenced by the adolescent–father relationship and vice-versa. Furthermore, associations between patterns of adolescent–parent divergence and convergence in parenting perceptions with substance use and aggressive behavior outcomes have not yet been assessed.

### The current study

In this study, we used a large sample of two-parent families with adolescent children to address two central research aims. Aim 1 was to identify theoretically meaningful constellations (i.e., profiles) of mother, father, and adolescent reports of parental warmth and their degrees of divergence or convergence. We expected that adolescents, on average, would endorse lower levels of parental warmth compared to parents’ self-reports (e.g., Hou et al., 2020). We expected to identify multiple profiles of adolescent–parent warmth discrepancies in our LPA. Among the profiles, we expected to find a *convergent high warmth* profile in which parents and adolescents all reported high levels of warmth and a *convergent low warmth* profile in which family members rated generally low levels of warmth in the family, consistent with prior studies identifying family subgroups (e.g., Hou et al., 2018; Xia et al., 2020). In addition, we expected to find family subgroups reflecting a *positive adolescent*–*parent divergence*, in which adolescents reported higher parental warmth than parents self-reported, and *negative adolescent*–*parent divergence* a profile in which adolescents reported lower parental warmth than parents self-reported. Further, we anticipated additional subgroups to emerge that might reflect distinct mother–adolescent and father–adolescent patterns of discrepancies. For instance, a *negative adolescent*–*mother divergence* profile in which both parents report high levels of warmth but adolescents only converge on high father warmth, reporting average or low mother warmth, could be observed. Similarly, a *negative adolescent*–*father divergence profile* in which parents report high levels of warmth but adolescents only converge with mother reports could be observed. Because a multitude of such patterns could exist, we considered this analysis to be exploratory.

Aim 2 was to leverage our longitudinal data to evaluate whether adolescents from families with different constellations of parental warmth discrepancies in 6^th^ grade had different levels of positive well-being, substance use initiation, and maladjustment outcomes three years later, at the end of 9^th^ grade. We expected that membership in profiles in which parents and adolescents converge on low levels of parental warmth would be associated with the greatest risk for maladjustment, substance use risk, and diminished positive well-being, followed by membership in profiles characterized by negative divergence in one or both adolescent–parent dyads. We expected that adolescent–parent convergence on high levels of parental warmth, as well as positive adolescent–parent divergence, would be associated with relatively lower risk for maladjustment, substance use initiation, and diminished positive well-being.

## Method

### Participants

Participants were a subsample from the PROmoting School-community-university Partnerships to Enhance Resilience (PROSPER) project, which is a three-component community-university partnership delivery system for evidence-based prevention intervention programs (Spoth et al., 2004). The project involved 28 school districts from rural Iowa and Pennsylvania and used a two-cohort sequential design. Initial eligibility criteria for communities in the study were (1) school district enrollment between 1300 and 5200 students and (2) at least 15% of students eligible for free or reduced-cost school lunches. Adolescents and their families were first assessed when they were in 6^th^ grade. Community teams selected a sequence of two interventions from a menu of evidence-based programs to be delivered using the PROSPER intervention model, consisting of a family-focused intervention to be delivered in the 6^th^ grade year followed by a school-based intervention during 7^th^ grade. Following initial assessment in the Fall of adolescents’ 6^th^ grade year, assessments were conducted annually until the spring of 12^th^ grade. The current study utilizes assessments from the Fall of 6^th^ grade (Wave 1) and the Spring of 9^th^ grade (Wave 5).

A random sample of 2,267 families from the second cohort were invited to participate in-home family assessments. Of these, 980 (43%) agreed to participate and completed assessments, which included a family interview and separate written questionnaires completed by the adolescent and each parent. The current study represents the subsample of two-parent, mother–father families (and parent–stepparent families) from this in-home sample (*N* = 687). Adolescents (52% girls) were on average 11.27 years old (*SD* = 0.49) at the 6^th^ grade assessment and 14.91 years old (*SD* = 0.45) at 9^th^ grade assessment. Adolescents identified their race as white (88.9%), Latino or Hispanic (6.3%), Black / African American (1.2%), Asian (0.6%), or Other (1.9%; 1.2% were missing data on race). Mothers’ ages ranged from 25 to 60 years old (*M* = 38.9, *SD* = 5.6) at the first assessment and 29–64 years old (*M* = 42.9, *SD* = 5.5) at the 9^th^ grade assessment; fathers were between 22 and 66 years old (*M* = 41.2, *SD* = 6.8) at the first assessment and 25 and 69 years old (*M* = 45.2, *SD* = 6.7) at the 9^th^ grade assessment. Parents described their race as white (79.3%), Latino or Hispanic (5.4%), Black / African American (1.8%), Native American / American Indian (0.2%), Asian (0.2%), and Other (0.9%; 1.0% were missing data on race). Family annual income was assessed at baseline and ranged from $9 to $362,661 (Median = $55,000). By the 9^th^ grade timepoint, 75.5% of the families remained in the study (n = 519).

### Measures

#### Parental warmth (6^th^ grade)

Parental warmth was measured for mothers, fathers, and adolescents in the Fall of 6^th^ grade. Mothers and fathers completed surveys reporting on their perceived warmth toward the adolescent. Adolescents completed the same items, reporting on each parent’s warmth toward them. Parents responded to three items from the Affective Quality of the Relationship Scale (AQRS; Spoth et al., 1998). All items asked about their parental warmth in the last month. Items included, “*During the past month, when you & your child have spent time talking or doing things together, how often did you* .. . ” (1) *Let this child know you really care about him/her,*” (2) “*Let this child know that you appreciate him/her, his/her ideas, or the things he/she does,*” and (3) “*Act loving and affectionate toward him/her.*” Items were rated on a 7-point Likert scale from 1 [always] to 7 [never] and were recoded such that higher numbers indicated higher levels of warmth before an average score was calculated. Scale reliability was acceptable for both mother (*α* = .87) and father (*α* = .89) self-reports.

Adolescents responded to the same three items about each parent, which were re-worded for adolescent reports. For instance, “*During the past month when you and you mom have spent time talking or doing things together, how often did your mom let you know she really cares about you?*” Items were rated on a 7-point Likert scale from 1 [always] to 7 [never] and were recoded such that higher numbers indicated higher levels of warmth before an average score was calculated. Scale reliability was acceptable for adolescents’ reports of mothers’ (*α* = .83) and fathers’ warmth (*α* = .89).

#### Adolescent positive well-being (9^th^ grade)

Adolescents’ happiness and life satisfaction were indicators of positive well-being in 9^th^ grade. Happiness was measured as the average of adolescents’ responses to the four-item subjective happiness scale (*α* = .80; Lyubomirsky & Lepper, 1999). Each item consisted of a sentence stem followed by a Likert scale from 1 to 7 with anchors that completed the stem. For instance, the stem “*In general, I consider myself,*” was followed by anchors “*not a very happy person*” (1) and “*a very happy person*” (7).

Life satisfaction in 9^th^ grade was measured as the average of adolescents’ responses to five items from the Mental Health Inventory-38 (*α* = .90; Veit & Ware, 1983). Items assessed adolescents’ life satisfaction across the past month, including items such as, “*During the past month, how much of the time have you generally enjoyed the things you do,*” and “*During the past month, how much of the time has your daily life been full of things that were interesting to you?*” Items were rated on a scale from *all of the time* (1) to *none of the time* (6) and were scored such that higher values indicated greater life satisfaction.

#### Adolescent maladjustment (9^th^ grade)

Adolescents’ maladjustment was assessed in the Spring of 9^th^ grade. Adolescents self-reported their aggressive behavior using 20 items from the aggressive behavior subscale of the Youth Self-Report (*α* = .87; Achenbach & Edelbrook, 1991). Adolescents were asked to report how true each behavior was for them, such as “*I argue a lot*” and “*I disobey at school.*” Items were scored either 0 [not true], 1 [somewhat or sometimes true], or 2 [very true or often true] and coded such that higher scores indicated more aggressive behavior before an average score was calculated.

Adolescents reported on their own anxiety and depressive symptoms using the 14 items from the anxious/depressed subscale of the Youth Self Report (*α* = .89; Achenbach & Edelbrook, 1991). Items *included* “*I am unhappy, sad or depressed*” and “*I worry a lot.*” Responses used the same coding scheme as the aggressive behavior scale and were coded such that higher values indicated more anxiety and depressive symptoms before an average score was calculated.

#### Adolescent substance use (9^th^ grade)

Adolescents self-reported their alcohol and marijuana use in 9^th^ grade. Adolescents reported alcohol use with the item, “*Have you ever been drunk from drinking alcohol?*” They also reported on marijuana use initiation using the item, “*Have you ever smoked marijuana (grass, pot) or hashish (hash)?*” Each item had response options “yes” and “no,” and was coded dichotomously so that 0 indicated no and 1 indicated yes.

### Analytic strategy

In preparation for the main analyses, we calculated a parental warmth discrepancy score for each adolescent–parent dyad by subtracting the mother or father’s self-reported warmth from the adolescent’s report of that parent’s warmth. Therefore, positive discrepancy scores indicate adolescents had a relatively higher perception of parental warmth than that parent, and negative scores indicate adolescents had a relatively lower perception of parental warmth than the parent. Correlations and descriptive statistics for each study variable are presented in Table 1.

To address Aim 1, identifying theoretically meaningful patterns of mother, father, and adolescent perceptions of parent warmth in 6^th^ grade, we first conducted a latent profile analysis (LPA) with four indicators from the 6^th^ grade assessments: adolescent report of mother’s warmth, adolescent report of father’s warmth, adolescent–mother discrepancy score, and adolescent–father discrepancy score. Mother and father self-reported warmth indicators were not included in the model, as this information is redundant with the other indicators; specifically, mother and father scores can be calculated from corresponding adolescent scores and discrepancy scores (De Los Reyes et al., 2013; Laird & De Los Reyes, 2013). LPA is a data-driven approach that organizes the configurations of the selected manifest indicators into a typology of mutually exclusive and exhaustive latent profiles. Families that are grouped into the same profile are therefore similar to each other to the extent that their observed scores on the selected indicators are assumed to represent the same probability distributions (Vermunt & Magidson, 2002). The LPA results in membership probabilities that describe the distribution of the sample across the profiles (i.e., profile prevalence), and item-response means and variances that describe the averages and distributions of the indicators within each profile. We based our selection of a model on three aspects: model identification and stability, statistical fit criteria, and a consideration of theoretical interpretability of profiles. The two most common statistical fit criteria for selecting a model are Akaike’s Information Criterion (AIC; Akaike, 1974) and Bayesian Information Criterion (BIC; Schwartz, 1978), which when minimized indicate a more parsimonious model. Additional fit criterion are the model entropy (Celeux & Soromenho, 1996), which indicates the profile separation (e.g., how distinct profiles are form each other), and the bootstrap likelihood ratio test (BLRT; McCutcheon, 1987; McLachlan & Peel, 2000), which indicates whether the current model provides significantly better fit with the data compared to the previous model with one fewer profiles. In addition to optimizing on these fit criteria, the selected model should have profiles that are theoretically meaningful and distinct from each other.

In a second step of Aim 1, we tested associations between latent profile membership and mother and father self-reported warmth as same-timepoint outcomes of profile membership using the BCH approach in Mplus (Bakk & Vermunt, 2016). Effects of profile membership in these models revealed whether certain profiles were characterized by relatively higher and lower levels of mother- and father-reported warmth. This was done to facilitate interpretation of the degree of convergence and divergence between adolescent and parent perceptions of warmth specific to each profile. The BCH approach classifies individuals to profiles based on posterior probabilities, and then adjusts the outcome analysis for classification error. This approach is considered best practice for predicting outcomes from latent profile membership (Bakk & Kuha, 2021). Effects of profile membership on parental warmth are expressed as pairwise differences between profiles in the means of the parental warmth.

Aim 2 was carried out in two steps. In both steps, we used profile membership to predict outcomes using linear and logistic regression based on modal class assignment with measurement-error weighting (Bakk & Vermunt, 2016; using BCH weights in Mplus). First, we assessed whether latent profile membership was differentially associated with adolescents’ adjustment problems and substance use in 9^th^ grade by regressing 9^th^ grade outcomes onto 6^th^ grade profile membership and the 6^th^ grade level of the outcome variable as a control. Effects of profile membership on outcomes are expressed as the association with each outcome for each profile compared to a reference profile. The reference profile can be respecified to other profiles to facilitate interpretation, without affecting the overall test of association. When considering substance use initiation outcomes (drunkenness and marijuana use), adolescents who endorsed already having been drunk (*n* = 1) or used marijuana (*n* = 1) at 6^th^ grade were excluded from each respective analysis, so that substance use initiation could be modeled more directly. Because we were also interested in whether profile membership was associated with unadjusted levels of clinically relevant outcomes, we also conducted a supplementary second step analyzing associations between profile membership and 9^th^ grade outcomes that did not control for initial levels of each outcome.

An additional consideration was whether to control for exposure to the school- and family-based interventions in outcomes analyses. Both interventions occurred after the LPA indicators were measured and were not evenly distributed in the current sample, because those in the current sample opted into home observation visits where the current data were collected. As an initial test, we examined associations of profile membership with the school-based intervention condition (which was randomized) and with exposure to the family-based intervention (which was voluntary). Profile membership was not significantly associated with being in the school-based intervention condition (*χ*^2^ = 4.09, *p* = .395) or exposure to the family-based intervention (*χ*^2^ = 2.63, *p* = .622). Additionally, parent and adolescent reports of parental warmth were not significantly different between intervention conditions (*t-*values from .19 to .82, all *p-*values > .05) or between those who did and did not chose to attend the family-based intervention sessions (*t-*values from .02 to 1.08, all *p-*values > .05), suggesting that those who participated in the interventions did not come from families with specific patterns of parental warmth reports. Adding intervention exposure, which occurred after the LPA indicators were measured, would introduce endogeneity and complexity to the longitudinal models, so we opted to not include these variables.

## Results

### Aim 1: identification of latent profiles

We considered latent profile solutions with 1 through 10 profiles. Fit criteria from each model are shown in Table 2. Increasing entropy values and significant BLRTs suggested that each model with additional profiles fit better than the last. The reductions in AIC and BIC were notably reduced in the four-profile solution and above. However, closer examination of the profiles revealed that new profile in solutions with 6 or more profiles did not provide meaningfully distinguishable groups. Therefore, the best fitting and most substantively meaningful models were those with 4 and 5 profile solutions. After detailed examination, the five-profile solution was selected due to a slightly better model fit and the fact that the emergent profile (which was not present in a four-profile solution) reflected a pattern of adolescent–parent divergence that has not been fully explored in prior work. In the 5-profile solution, two of the latent profiles were small in proportion to the full sample; however, they were stable across solutions (emerging in the three- and four-profile solutions and remaining with similar size and interpretation across the five-profile solution and beyond). Furthermore, the smallest profile was the most consistent with a traditional interpretation of risk conferred by adolescent–parent divergence, in the sense that this profile reflected families in which adolescents had significantly lower perceptions of parental warmth compared to parents. The emergent profile in the five-profile model was also small but had an interpretation that was meaningfully different from the existing profiles. Additionally, this new profile remained stable into the six-profile solution as well. Therefore, the selected model was the five-profile solution.

**Figure 1.**
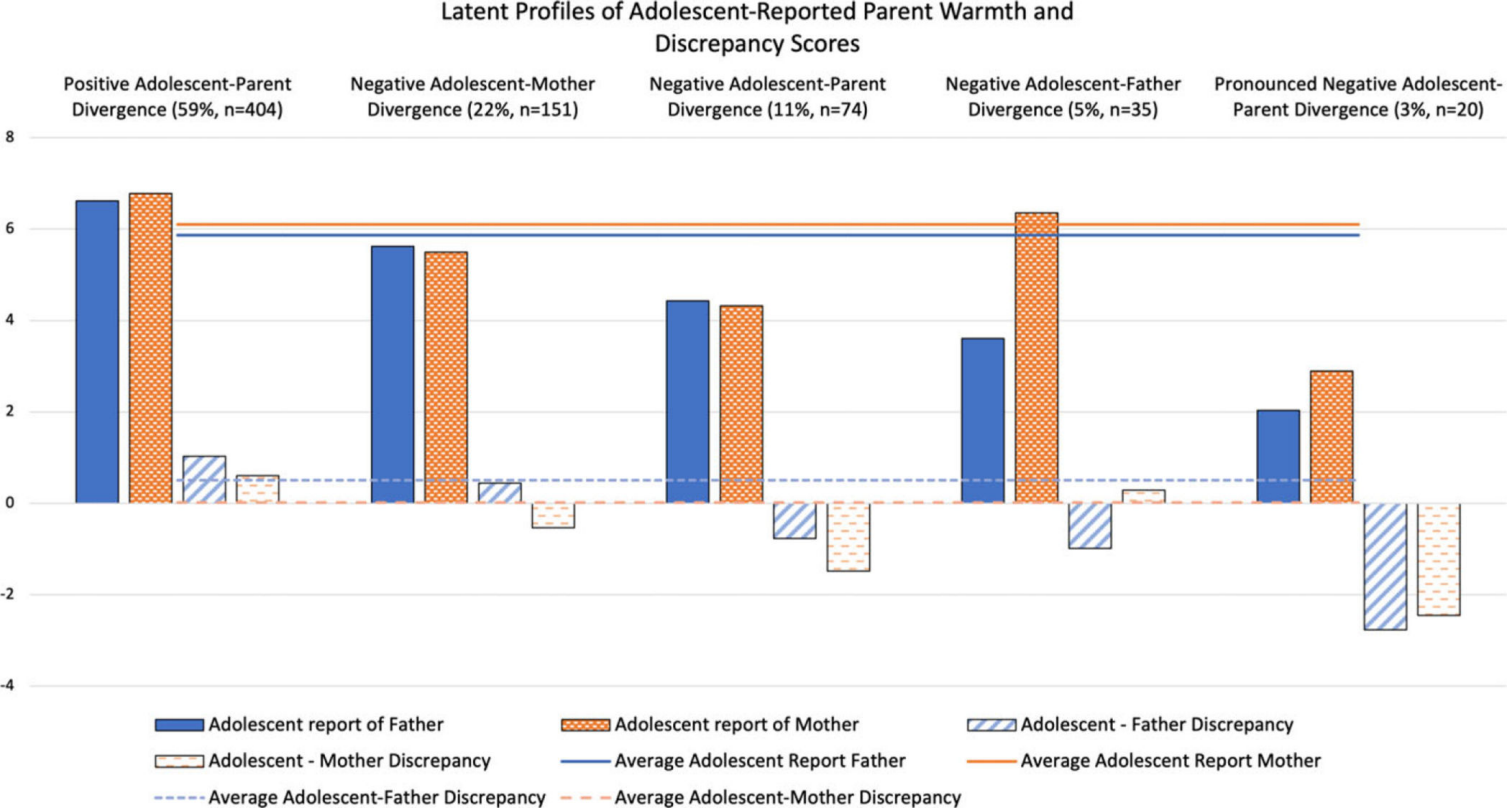
Latent profiles of adolescent-reported parent warmth and discrepancy scores.

The within-profile item means and their comparison to the sample means are shown in Table 3 and displayed in Figure 1. Adolescent–parent discrepancy scores were on average positive, indicating that adolescents on average had higher reports of parental warmth compared to parents’ self-reports (*M*_adolescent–father_ = 0.50, *SD*_adolescent–father_ = 1.36; *M*_adolescent–mother_ =0.02, *SD*_adolescent–mother_ = 1.18). Therefore, positive profile-specific estimates indicate positive adolescent–parent divergence in which adolescents have higher perceptions of parental warmth compared to parents, and negative estimates indicate divergence in which adolescents have lower perceptions of parental warmth compared to parents. Within-profile item means for discrepancy scores were compared to both zero and to the sample average for that discrepancy score, as shown in Table 3. The largest profile was a *Positive Adolescent*–*Parent Divergence* (59%) profile, in which adolescent-reported warmth was higher than the sample average and higher than parent reports. The second profile was a *Negative Adolescent*–*Mother Divergence* (22%) profile, in which adolescent reports of maternal warmth were below average and were lower than mothers’ self-reports, but adolescent reports of paternal warmth and fathers’ self-reported warmth converged around the average. The third profile, *Negative Adolescent*–*Parent Divergence* (11%), was haracterized by negative adolescent–parent discrepancies in both adolescent–mother and adolescent–father relationships. The fourth profile was characterized by *Negative Adolescent*–*Father Divergence* (5%), in which adolescent reports of paternal warmth were lower than average and relatively lower than fathers’ self-reported warmth, whereas adolescent reports of mother’s warmth were not different from the average or from mother self-reports. Finally, a *Pronounced Negative Adolescent*–*Parent Divergence* (3%) profile was observed in which adolescent reports of parental warmth were extremely low and much lower than parent reports, consistent with a traditional interpretation of risk conferred by adolescents’ more negative perceptions of family functioning.

In the second step of Aim 1, we treated mother- and father-reported warmth as outcomes of profile membership to obtain profile-specific estimates of parent self-reported warmth. The results showing pairwise comparisons of parent warmth across profiles are presented in Table 3. The *Positive Adolescent*–*Parent Divergence* profile was characterized by higher paternal warmth than all other profiles (*χ*^2^values from 6.58 to 12.89, *p* values from .01 to < .001), and higher maternal warmth than the *Negative Adolescent*–*Parent Divergence* (*χ*^2^= 12.16, *p* < .001) and the *Pronounced Negative Adolescent*–*Parent Divergence* (*χ*^2^ = 9.89, *p* = .002) profiles. The *Negative Adolescent*–*Father Divergence* profile also showed lower levels of paternal warmth than the *Negative Adolescent*–*Mother Divergence* (*χ*^2^ = 4.37, *p* = .037) and *Negative Adolescent*–*Parent Divergence* (*χ*^2^= 4.54, *p* = .033) profiles. The *Pronounced Adolescent*–*Parent Divergence* profile was also characterized by lower maternal warmth than the *Negative Adolescent*–*Mother Divergence* (*χ*^2^= 6.13, *p* = .013) and *Negative Adolescent*–*Father Divergence* (*χ*^2^= 5.83, *p* = .016) profiles.

We conducted a supplementary post hoc analysis to determine whether profile-specific estimates for each indicator were not only statistically different from the sample mean, but to the means of the other profiles. We did so by re-running the LPA with indicator means centered around profile-specific means for each profile in turn. For instance, we began by centering the mean of each indicator at the within-profile mean estimate for that indicator in Profile 5, such that instead of within-profile means being compared to the sample mean, they were being compared to the means of Profile 5. The results, presented in Supplemental Table 1, reflect that nearly all profile-specific means were different from all other profiles at a *p* < .05 level. This adds to our confidence that the chosen LPA solution represents five distinct profiles of adolescent and parent perceptions of parental warmth.

### Aim 2: longitudinal associations between latent profiles and outcomes

To address Aim 2, we conducted a prospective analysis of differential associations between profile membership in 6^th^ grade and adolescent positive well-being, maladjustment, and substance use outcomes in 9^th^ grade (Table 4). In models with continuous dependent variables, we controlled for 6^th^ grade levels of each dependent variable. In all models, initial levels of the dependent variable significantly predicted 9^th^ grade levels (see Table 4). For binary dependent variables (drunkenness and marijuana initiation), those who had initiated the behavior by 6^th^ grade (*n* = 1 for both outcomes) were dropped from the analyses so that initiation of the behavior could be modeled. Results are interpreted against a reference profile. We respecified the reference profile so we could interpret prediction of each outcome for membership from each profile compared to each other profile, starting with the *positive adolescent*–*parent divergence* profile. Results are displayed in Table 4, with each panel representing a different rotation of the same overall significance test. Some comparisons are redundant (e.g., when Profile 3 is the reference profile, the comparison between Profiles 1 and 3 were already displayed when Profile 1 was the reference profile), and these are not shown in the table.

We found profile-specific differences in 9^th^ grade life satisfaction, odds of having been drunk, and odds of having used marijuana. Specifically, adolescents from families characterized by the *negative adolescent*–*mother divergence* profile had significantly lower levels of life satisfaction compared to those in the *positive adolescent*–*parent divergence* profile (*β* = −0.34, *p* = .005) and those in the *negative adolescent*–*parent divergence* profile (*β* = −0.39, *p* = .039), holding 6^th^ grade life satisfaction constant. For substance use initiation outcomes, we found that adolescents in the *pronounced negative adolescent*–*parent divergence* profile had significantly higher odds of initiating marijuana use by 9^th^ grade compared to those in the less extreme *negative adolescent*–*parent divergence* profile (OR = 10.32, 95% CI [1.69, 63.11]). Additionally, adolescents in the *pronounced negative adolescent*–*parent divergence* were 3.22 times more likely to have initiated marijuana use (95% CI [1.17, 8.84]) and 2.62 times more likely to have been drunk (95% CI [1.07, 6.43]) compared to those in the *positive adolescent*–*parent divergence* profile.

### Supplemental analysis: unadjusted outcomes across profiles

Our final step of analysis was to examine whether profile membership early in adolescence could provide a strong signal for risk of outcomes in later adolescence. We therefore estimated models that predicted each outcome from latent profile membership but did not adjust for 6^th^ grade levels of the outcome. The results of this analysis are presented in Supplemental Table 2 in the supplemental materials. Overall likelihood ratio tests indicated that profile membership in 6^th^ grade was significantly associated with life satisfaction (*χ*^2^ = 22.84, *p* < 0.001) and aggressive behavior (*χ*^2^ = 10.89, *p* = 0.028) in 9^th^ grade. As in the adjusted models, pairwise comparisons suggested that adolescents from families characterized by *positive adolescent*–*parent divergence* had higher levels of life satisfaction than those in the *negative adolescent*–*mother divergence* profile (*χ*^2^ = 16.17, *p* < 0.001), but also higher life satisfaction than those in the *negative adolescent*–*father divergence* profile (*χ*^2^ = 6.16, *p* = 0.013). Additionally, those in the *positive adolescent*–*parent divergence* profile exhibited diminished risk for aggressive behavior compared to those in the *negative adolescent*–*mother divergence* (*χ*^2^ = 7.27, *p* = 0.007) and *pronounced negative adolescent*–*parent divergence* profiles (*χ*^2^ = 4.74, *p* = 0.029).

## Discussion

Parents and adolescents often have different perceptions of family relations, such as parenting practices, an issue that is often overlooked in the family and developmental literature. Multi-informant studies have paved the way for the examination of the nature and implications of adolescent–parent informant discrepancies. In the current study, we used LPA to identify five profiles of convergence and divergence in mother, father, and early adolescent reports of parental warmth. Despite family scholars’ theoretical emphasis on family *wholism*—the idea that family subsystems cannot be understood as merely component parts, calling for a more complete conceptualization of the system beyond reduction to individual dyads (Minuchin, 1985)—few studies of informant discrepancies have used methods that incorporate both mother–adolescent and father–adolescent reports simultaneously.

### Aim 1: latent profiles of mother, father, and adolescent perceptions of parental warmth

Our LPA identified five profiles that supported our hypotheses that some families would exhibit generally high warmth, some would be generally low in warmth, and other families would exhibit adolescent–parent divergence in perceptions of one parent, but not in the other parent. Over half of families (59%) fit into a *positive adolescent*–*parent divergence* profile in which adolescents had more positive perceptions of parenting relative to the sample average and to their parents. Specific patterns of unbalanced adolescent–parent divergence were also prominent, with 22% of families characterized by *negative adolescent*–*mother divergence* (adolescents having a more negative perception of maternal warmth than mothers, but converging reports with fathers), and 5% of families characterized by *negative adolescent*–*father divergence* (adolescents having a more negative perception of paternal warmth than fathers, but converging reports with mothers). Additionally, two profiles were characterized by patterns of negative adolescent divergence in perceptions from both parents, with one characterized by moderate *negative adolescent*–*parent divergence* (11%) and one reflecting more extreme or *pronounced negative adolescent*–*parent divergence* (3%), in which adolescents’ perceptions of parental warmth were extremely low as well as being lower than parent’ self-reported warmth. The diversity in observed patterns lends support to the calls from family systems scholars for more wholistic conceptualizations of family relationships and informant discrepancies (Goodman et al., 2010; Minuchin, 1985). Consistent across all profiles was that patterns marked by adolescent–parent divergence seemed to be driven by subsets of adolescents who had either more positive or more negative perceptions than the sample mean, whereas parents’ self-reports hovered closer to the sample mean across profiles.

Our findings align with other person-centered analyses that document the occurrence of positive adolescent–parent discrepancies, although the prevalence of this pattern was higher in the current study compared to others (Hou et al., 2018; Rote & Smetana, 2016). Of note, adolescents characterized by this pattern also reported parental warmth that was higher than average, in addition to being higher than parental reports. Prior studies, especially those using variable-centered approaches, document a greater likelihood of adolescents’ perceptions of family functioning being more negative than their parents (e.g., Hou et al., 2020). However, other studies have documented that positive adolescent–parent divergence in perceived parenting may be especially common early in adolescence and that adolescent–parent perceptions become more similar over time (Mastrotheodoros et al., 2018; Gniewosz et al., 2023). Therefore, the prevalence of positive adolescent–parent discrepancies in the current study could be due to the young age of the adolescents. Additionally, the high proportion of families characterized by positive adolescent–parent divergence suggests that perhaps in previous studies, observations that adolescents on average had more negative perceptions of the family than parents could have been driven by small subgroups of families in which adolescents had extremely negative perceptions of family functioning. Indeed, the *Pronounced Adolescent*–*Parent Divergence* profile observed in the current study was the smallest in size but exhibited the largest magnitude adolescent–parent discrepancies of any profile, which could disproportionately affect the overall sample means of discrepancy scores. This example further illustrates the utility of family-centered approaches like LPA to better reflect the occurrence of patterns of adolescent–parent convergence and divergence.

About one in four families (27%) in this sample were characterized by a pattern of unbalanced divergence in which adolescents’ perceptions of parenting were convergent in one parent–adolescent dyad but divergent in the other. In each case, adolescents reported that one parents’ warmth was average while the other was lower than average. This finding may reflect each parent’s unique role in parenting their adolescent; warmth conferred by one parent may be different in its behavioral manifestations than warmth conferred by the other parent (Padilla-Walker et al., 2016; Palkovitz et al., 2014), and these may have different implications for perceived warmth by adolescents. Such patterns of unbalanced divergence have not been sufficiently reviewed in the literature, because most studies evaluate adolescent–mother and adolescent–father dyads separately. The prevalence of unbalanced divergence patterns suggests that methods that cannot accommodate multiple adolescent–caregiver relationships within the same models will fail to result in accurate depictions of the lived experiences of adolescents in which caregiver relationships exist in the context of other subsystems and the family as a whole. Therefore, family-centered methods like LPA represent a fruitful statistical approach for future studies of multi-informant patterns of perception, and these should be capitalized on for exploring the potential for unbalanced adolescent–parent divergence patterns across other domains of family functioning.

Contrary to our hypotheses, our findings did not reveal clear patterns of adolescent-parent convergence on high or low levels of parental warmth. Rather, even families in which adolescents and parents both reported high degrees of parental warmth were characterized by a degree of positive adolescent–parent divergence. Similarly, for profiles in which one or both parents reported slightly lower than average warmth, adolescents’ reports of that parent were still relatively lower than parents’ self-reports, suggesting that adolescents’ perceptions of parental warmth may be similar to but more extreme than parents’ own views. In the two profiles wherein adolescent–parent convergence on parental warmth was observed in one of the adolescent-parent dyads, it was in the context of divergence in the other dyad (i.e., the *negative adolescent*–*mother divergence* and *negative adolescent*–*father divergence* profiles). This contrasts with prior person-centered approaches for studying adolescent–parent informant discrepancies in which convergent groups were observed (De Los Reyes et al., 2010; Hou et al., 2018; Rote & Smetana, 2016). However, our approach differed from these previous studies by incorporating both adolescent–parent dyads simultaneously. The lack of an overall pattern of adolescent–parent convergence in this study suggests that parental warmth may be one family construct that is particularly prone to divergent perspectives; it may be that adolescents’ and parents’ individual well-being and perceptions of other family relationships can heavily influence their perceptions of parental warmth, resulting in a prevalence of discrepancies. Therefore, potential family and individual contextual factors that may influence or even bias adolescent and parent perceptions of parental warmth should be examined as correlates of patterns of convergence and divergence in reports of parental warmth.

### Aim 2: associations of profile membership with positive well-being, substance use, and maladjustment

Prior work has established links between adolescent–parent discrepancies in parenting quality and negative developmental outcomes (Gniewosz et al., 2023; Guion et al., 2009; Hou et al., 2018; Van Petegem et al., 2020). However, the developmental implications of patterns of positive adolescent–parent divergence, as well as unbalanced divergence from only one parent or the other, have not been widely explored. We observed that when early adolescents perceived higher levels of parental warmth than average, which were also higher relative to their parents, they exhibited higher levels of life satisfaction later in adolescence (9^th^ grade) compared to those whose perceptions negatively diverged from mothers only or fathers only, controlling for initial levels. Adolescents’ perceptions of positive parenting early in adolescence has been associated with later life satisfaction (Leung et al., 2004; Teubert & Pinquart, 2011), perhaps because adolescents who perceive positive and supportive family relationships can better develop a positive or growth mindset that promotes positive wellbeing later (Jach et al., 2018; Mueller et al., 2017). Additionally, adolescents who perceive more supportive parenting earlier in adolescence are better protected from declines in the adolescent– parent relationship across adolescence (Laursen et al., 2010). In light of prior work, our finding suggests that although adolescents’ more positive view of parenting may wane across the developmental period (Mastrotheodoros et al., 2018), an early positive perception of parenting may still serve to promote positive well-being into later years.

Negative divergence in the adolescent–mother dyad (but not adolescent–father dyad) was the second most common pattern observed in this study, and this pattern was associated with risk for diminished positive well-being. Interestingly, adolescents characterized by *negative adolescent*–*mother divergence* reported that the amount of warmth they received from mothers and fathers was about the same. However, their reports were lower than mothers’ own reports which were much closer to average. It may be that for mother–adolescent dyads in particular, societal standards of mothering may play a role in promoting unbalanced divergence. That is, when considering parent warmth, adolescents may be comparing their mothers not to their fathers, but to a higher standard of maternal warmth that is set by outside influences like popular media as well as other mother figures they encounter, such as their peers’ parents. This may lead many adolescents to report that their mothers’ warmth is lower than average, while they perceive fathers who provide roughly the same amount of warmth as meeting parenting expectations. In contrast, parents are likely reflecting more on their own parenting intentions when asked about parenting behaviors (Feldman et al., 1989), which may yield relatively higher reports of warmth. The association of membership in this profile with diminished adolescent well-being implies that despite perceiving similar levels of warmth from fathers and mothers, adolescents’ relatively lower perceptions of maternal warmth specifically may pose a threat to well-being.

We also found that adolescents in the *pronounced negative adolescent*–*parent divergence* profile, who perceived much lower parental warmth on average and compared to their parents, were over ten times more likely to have used marijuana by 9^th^ grade compared to those whose perceptions were low to a less extreme degree. In addition, adolescents in the *pronounced negative adolescent*–*parent divergence* profile were over twice as likely to have been drunk and to have used marijuana by 9^th^ grade compared to those whose perceptions of parenting were more positive than parents’ perceptions and the sample average. Prior work has documented within-family linkages between profiles of parenting behaviors and adolescent substance use (Choi et al., 2017; Koning et al., 2012; Xia et al., 2020), and the current study emphasizes the importance of including both levels and degree of divergence in adolescent–parent perceptions to comprehensively assess risk. These finding also support prior work that posits that adolescents’ more negative perceptions of parenting may be normative at a low level but pose a developmental risk at extreme levels (Carlson et al., 1991; Feinberg et al., 2000). This is particularly evident in that mother and father average self-reported warmth did not differ between the *negative adolescent*–*parent divergence* and *pronounced negative adolescent*–*parent divergence* profiles, suggesting that the extreme negative divergence of adolescent perceptions of parenting compared to parent perceptions may signal elevated risk for substance use. In contrast, having more positive perceptions of parenting compared to parents, combined with being higher than average, seemed to serve a protective role against substance use initiation.

Finally, results from unadjusted longitudinal models additionally indicated that adolescents who perceived higher warmth overall and compared to parents were at diminished risk for aggressive behavior compared to those who perceived more pronounced low parental warmth than parents, as well as those who perceived lower parental warmth compared to their mothers but not fathers. In prior studies applying person-centered methods, adolescents’ problem behavior has been associated with their under-reporting of disclosure and parental knowledge compared to parents (Rote & Smetana, 2016). Parental monitoring, warmth, and aggressive behavior are interconnected. Adolescents are more willing to disclose information when parental warmth is higher, and child disclosure could in turn promote parental warmth (Branje, 2018; Klevens & Hall, 2014). Effective parental monitoring and youth disclosure have been related to less aggressive behavior (Brendgen et al., 2001; Lee & Randolph, 2015; Yang et al., 2022), and aggressive behavior has in turn been related to poorer monitoring (Yang et al., 2022). Considered within the findings of the current study, which suggest a link between specific adolescent–parent perceptions of warmth and adolescents’ aggressive behavior, this body of work implies a need for a better understanding of the direction of relationships between parental warmth and adolescents’ aggression. It is possible that pronounced divergent perspectives of warmth represent the beginning of a process by which parents who struggle to provide warmth are also less likely to be effective in monitoring and knowledge of their adolescent’s activities, which allows aggressive behaviors to develop. Alternatively, some adolescents may have early predispositions toward aggressive behavior, including having more aggressive parents, which make parental warmth less likely (McCloskey & Lichter, 2003; Piquero et al., 2012). This directionality issue is compounded by the fact that little is known about how informant discrepancies may change across key developmental periods (De Los Reyes et al., 2023a). Therefore, future studies should explore potential bidirectional effects between adolescent–parent convergence and divergence in reports of parental warmth and adolescents’ aggressive behaviors or other precursors of aggression, across this developmental period.

### Implications for research and practice

This study has revealed new information about how adolescent–parent divergence in reports of parenting may signal meaningful risk or protection for adolescent developmental outcomes. Pronounced negative adolescent divergence from one or both parents in reports of parental warmth predicted risk for diminished positive well-being, and pronounced negative divergence further signaled risk for early alcohol and marijuana use initiation, whereas positive divergence and less extreme negative adolescent–parent divergence served more of a protective role. Given the potential persistence of adolescent well-being and substance use problems into adulthood (Grant & Dawson, 1997; McLeod et al., 2016; Odgers et al., 2008; Richards & Huppert, 2011), family practitioners could consider ways in which to identify patterns of adolescent–parent divergence that are extreme or are inconsistent across parent–adolescent dyads, in addition to generally low adolescent ratings of warmth, early in adolescence. Including adolescents in family-based parenting interventions could improve effectiveness (Van Ryzin et al., 2016) and may reveal new insights into the processes that drive each family member’s perceptions of parenting and other family contextual factors. Although only a few correlates of profiles of adolescent–parent convergence on parenting quality have been assessed (e.g., Rote & Smetana, 2016), they suggest that factors such as adolescent behavior problems as well as parent mental health and well-being may influence or bias perceptions of parenting. However, little is known about how informant discrepancies manifest at the individual family level, which is where many treatment approaches are focused (De Los Reyes et al., 2023a). Therefore, understanding how both adolescent and parent individual factors influence perceptions in a given family could represent an upstream intervention approach to reducing informant discrepancies.

This study has added to a body of work suggesting pronounced negative adolescent–parent divergence, in conjunction with low parental warmth in general, as an early warning sign of future maladjustment and risky behavior problems for developing adolescents (Gniewosz et al., 2023; Guion et al., 2009; Hou et al., 2018; Van Petegem et al., 2020), and adds diminished positive wellbeing to this set of outcomes. Adolescents whose perceptions of parenting showed pronounced negative divergence from parents’ own views were at elevated risk for substance use initiation compared to those who showed less pronounced divergence. Previous work using LPA within the current study sample has shown that incorporating additional family relationship facets, such as parent–child conflict, may further elucidate risk for early adolescent substance use initiation (Xia et al., 2020). Combined, this work suggests that adolescents’ pronounced negative perceptions of the family compared to parents, combined with poorer family relationships as a whole, could represent an additional warning sign for later risky behaviors. Further explorations into what family contextual factors may drive divergence in specific domains should be a goal of future research in order to identify potential early intervention targets that could prevent negative divergence in adolescent–parent perceptions from arising or escalating to extreme degrees.

### Limitations and future directions

Despite its contributions, this study must be interpreted in light of its limitations. Several factors may limit the generalizability of this study, including that the study population was mostly white and from rural residences. Future studies should seek to replicate these findings among more racially and geographically diverse groups of families, especially given the small size of some of the identified profiles. Additionally, the structure of the families in this study was homogenously heterosexual, two-parent families who resided in the same home, such that mother–adolescent and father–adolescent dyadic relationships could be explored together. It remains less clear how patterns of adolescent–parent divergence characterize and function within single-parent families, sexual and gender minority parent families, and families in which children reside across multiple households. Considering the promising findings, methods that can accommodate additional family structures should be given special attention in future research around adolescent–parent convergence and divergence.

Additionally, Type I error can pose a problem in any analyses involving a multinomial predictor, as multiple pairwise tests are required to characterize its association with an outcome variable. Further, effects involving smaller profiles may be obscured in an overall likelihood ratio test. We did conduct an overall likelihood ratio test for each outcome by comparing nested models wherein the effect of profile membership on each outcome was constrained to be equal and then freely estimated, and we found a significant log-likelihood difference only for the life satisfaction outcome. Therefore, the pairwise comparisons for other outcomes (drunkenness and marijuana initiation) should be interpreted with caution. The exploratory nature of this analysis combined with small but clearly distinct profiles lead us to refrain from relying on only one statistical approach, but overall tests of association can be a useful benchmark by which to prioritize future research with larger and more diverse samples.

Furthermore, the longitudinal nature of this study is a strength, but little is known about how patterns of divergence and convergence in adolescent–parent perceptions may change from early to later adolescence (De Los Reyes et al., 2023a). Therefore, future work should explore potential changes in profile prevalence across adolescence, using methods like latent transition analysis. Similarly, models that examine longitudinal, within-family effects between adolescent–parent discrepancies and outcomes present another opportunity for assessing the meaning of informant discrepancies across this developmental period. For instance, random-intercept cross-lagged panel models represent a fruitful approach for harnessing multiwave longitudinal data and discerning temporal effects of discrepancies (e.g., Vrolijk et al., 2023). Although the aim of the current study was to identify unique patterns of mother, father, and adolescent convergence and divergence in perspectives, alternative models could shed light on the longitudinal, possibly reciprocal relationships between these specific typologies and adolescent outcomes across time. Despite being beyond the scope of the current study, such within-family approaches should be explored. Finally, despite the importance of parental warmth for a range of adolescent developmental outcomes, adolescent–parent convergence and divergence in other parenting dimensions should be explored, as divergence in specific domains may be uniquely associated with outcomes not explored in this study.

## Conclusion

This study used the family-centered method of latent profile analysis to uncover previously understudied patterns of divergence and convergence in mother–adolescent and father–adolescent perceptions of parental warmth. In particular, adolescents who perceived low parental warmth and had a dramatically more negative view of parental warmth compared to one or both parents were at elevated risk for diminished positive well-being later in adolescence, compared to adolescents who had a more positive perception of parental warmth. Families where adolescents had extremely negative perceptions of parental warmth compared to parents were a minority in this study, despite this pattern of divergence being the focus of previous work. Nonetheless, patterns of extreme negative adolescent–parent divergence seemed to confer additional risk for early alcohol and marijuana initiation, above and beyond perceived levels of parental warmth. These findings contribute to a body of work implicating extreme negative adolescent–parent divergence as a risk factor for adolescent development. We added nuance by assessing the two adolescent–parent dyads in tandem, revealing that specific patterns of unbalanced mother–adolescent and father–adolescent divergence were common and were uniquely associated with negative developmental outcomes. Future studies should work toward understanding the family context from which adolescent–parent divergence arises, and whether viable intervention targets are among these contextual factors. This will be an important next step to curbing the public health costs that occur as a result of adolescent psychopathologies.

## Supplementary Material

1

2

## Figures and Tables

**Table 1.** Pairwise correlations, sample sizes, and descriptive statistics

	1	2	3	4	5	6	7	8	9	10	11	12
1. A Report Father Warmth	–	680	605	681	605	681	514	515	521	514	513	513
2. A Report Mother Warmth	.65**	–	603	681	603	679	515	516	522	515	514	514
3. Father Self−Rep Warmth	.40**	.19**	–	607	605	604	467	468	472	467	466	466
4. Mother Self−Rep Warmth	.23**	.27**	.23**	–	604	681	517	518	514	517	516	516
5. A − F Discrepancy	.66**	.47**	−.42**	.04	–	604	465	466	470	465	464	464
6. A − M Discrepancy	.42**	.71**	−.00	−.48**	.40**	–	513	514	520	513	512	512
7. Wave 5 Happiness	.07	.12**	.07	.14**	.03	.01	–	518	514	514	516	516
8. Wave 5 Life Satisfaction	.15**	.14**	.06	.06	.11*	.09*	.68**	–	515	515	517	517
9. Wave 5 Drunkenness	−.06	−.06	−.04	.05	−.02	−.09*	−.11*	−.13**	–	517	514	514
10. Wave 5 Marijuana	−.07	−.00	−.07	.03	.01	−.02	−.15**	−.07	.47**	–	514	514
11. Wave 5 Anx / Dep	−.06	−.10*	−.02	.06	−.03	−.05	−.47**	−.43**	.16**	.10*	–	517
12. Wave 5 Aggressive Behavior	−.11*	−.12**	−.09*	.10*	−.01	−.04	−.32**	−.31**	.23**	.22**	.58**	–
*Mean*	5.86	6.10	5.40	6.08	0.50	0.02	5.34	4.79	0.19	0.09	0.26	0.30
*SD*	1.37	1.07	1.11	0.86	1.36	1.18	1.16	0.97	0.39	0.29	0.33	0.28

*Note: F* = Father; *M* = Mother; *A* = Adolescent. two−sided bivariate correlations were calculated; correlations are shown below the diagonal; sample size for each correlation is shown above the diagonal;

* *p* < .05,

** *p* < .01,

*** *p* < .001.

**Table 2.** Fit parameters of 1-through 10-profile latent profile solutions

No. of Profiles	No. of Free Parameters	Log-Likelihood	AIC	BIC	a-BIC	a-BIC Difference	Entropy	BLRT
1	8	−4321.54	8659.07	8695.30	8669.90	—	—	—
2	13	−3870.34	7766.67	7825.53	7784.26	−885.64	0.89	*p* < .001
3	18	−3709.35	7454.70	7536.20	7479.05	−305.21	0.89	*p* < .001
4	23	−3613.63	7273.25	7377.40	7304.37	−174.68	0.89	*p* < .001
**5**	**28**	**−3536.42**	**7128.84**	**7255.62**	**7166.72**	**−137.65**	**0.91**	***p* < .001**
6	33	−3474.33	7014.67	7164.09	7059.31	−107.41	0.91	*p* < .001
7	38	−3438.13	6952.25	7124.32	7003.66	−55.65	0.92	*p* < .001
8	43	−3401.98	6889.96	7084.66	6948.13	−55.53	0.93	*p* < .001
9	48	−3364.30	6824.59	7041.94	6889.53	−58.60	0.91	*p* < .001
10	53	−3335.23	6776.46	7016.44	6848.16	−41.37	0.92	*p* < .001

*Note:* AIC = Akaike information criterion; BIC = Bayesian information criterion; a − BIC = sample-size adjusted BIC; BLRT = Bootstrapped likelihood ratio test; Bold text indicates the selected model.

**Table 3.** Profiles of adolescent, mother, and father divergence and convergence in reports of parental warmth

		1	2	3	4	5
	Sample Mean (SD)	Positive Adolescent–Parent Divergence	Negative Adolescent–Mother Divergence	Negative Adolescent–Parent Divergence	Negative Adolescent–Father Divergence	Pronounced Negative Adolescent–Parent Divergence
*LPA Indicators*		(59%, *n* = 404)	(22%, *n* = 151)	(11%, *n* = 74)	(5%, *n* = 35)	(3%, *n* = 20)

Adol. Report of Father	5.87 (1.37)	6.62^^^	5.62	4.43^v^	3.61^v^	2.03^v^

Adol. Report of Mother	6.10 (1.07)	6.78^^^	5.50^v^	4.32^v^	6.36	2.89^v^

Adol.-Father Discrepancy	0.50 (1.36)	1.03^^^**	0.44*	−0.77^v^**	−0.99^v^**	−2.77^v^**

Adol.-Mother Discrepancy	0.02 (1.18)	0.60^^^**	−0.54^v^**	−1.49^v^**	0.29	−2.45^v^**

*Predicting Parent Self-Reported Warmth (6^th^ Grade)*						

Father Self-Reported Warmth^a^	5.41 (1.11)	5.62^2,3,4,5^ (0.06)	5.20^1,4^ (0.11)	5.23^1,4^ (0.14)	4.51^1,2,3^ (0.30)	4.88^1^ (0.27)

Mother Self-Reported Warmth^a^	6.08 (0.86)	6.19^3,5^ (0.04)	6.03^5^ (0.08)	5.78^1^ (0.11)	6.11^5^ (0.17)	5.35^1,2,4^ (0.26)

Adol. = Adolescent. Statistical significance is at *p* < .05.

^ Profile mean was significantly higher than sample mean.

v Profile mean was significantly lower than sample mean.

* Profile mean discrepancy score was different from 0 using statistical significance of *p* < .05.

** Profile mean discrepancy score was different from 0 using statistical significance of *p* < .01.

a Latent profile membership was an overall significant predictor of the outcome.

1 profile mean was significantly different from mean of Profile 1.

2 profile mean was significantly different from mean of Profile 2.

4 profile mean was significantly different from mean of Profile 4.

5 profile mean was significantly different from mean of Profile 5.

**Table 4.** Longitudinal associations between 6^th^ grade latent profiles and 9^th^ grade outcomes

	Happiness	Life Satisfaction	Aggressive Behavior	Anxious/Depressed	Been Drunk Ever	Marijuana Ever
	*β* (SE)	*β* (SE)	*β* (SE)	*β* (SE)	OR [95% CI]	OR [95% CI]
Intercept	3.25***(.33)	3.37*** (.29)	.15*** (.02)	.16*** (.02)	.18*** [.14, .21]	.09*** [.06, .11]

6^th^ Grade Level	.38*** (.05)	.29*** (.05)	.56*** (.05)	.33*** (.05)		

1. *Positive Adol-Parent Divergence*	*ref*	*ref*	*ref*	*ref*	*ref*	*ref*

2. Negative Adol-Mother Divergence	−.11 (.15)	−.34** (.12)	.03 (.03)	.06 (.05)	1.19 [.71, 2.01]	1.19 [.60, 2.36]

3. Negative Adol-Parent Divergence	.17 (.19)	.05 (.17)	−.01 (.04)	.04 (.05)	1.08 [.57, 2.06]	.31 [.07, 1.45]

4. Negative Adol-Father Divergence	.12 (.26)	−.30 (.19)	−.01 (.04)	−.03 (.06)	.86 [.29, 2.59]	.84 [.19, 3.74]

5. Pronounced Negative Adol-Parent Divergence	−.28 (.32)	−.12 (.22)	.05 (.07)	.01 (.11)	**2.62 [1.07, 6.43]**	**3.22 [1.17, 8.84]**

2. *Negative Adol-Mother Divergence*	*ref*	*ref*	*ref*	*ref*	*ref*	*ref*

3. Negative Adol-Parent Divergence	.28 (.22)	.39* (.19)	−.04 (.05)	−.02 (.07)	.91 [.42, 1.96]	.26 [.05, 1.37]

4. Negative Adol-Father Divergence	.24 (.28)	.05 (.22)	−.03 (.05)	−.09 (.07)	.72 [.22, 2.35]	.71 [.14, 3.49]

5. Pronounced Negative Adol-Parent Divergence	−.17 (.33)	.23 (.23)	.03 (.08)	−.04 (.11)	2.20 [.84, 5.77]	2.71 [.89, 8.24]

3. *Negative Adol-Parent Divergence*	*ref*	*ref*	*ref*	*ref*	*ref*	*ref*

4. Negative Adol-Father Divergence	−.05 (.30)	−.35 (.24)	.00 (.05)	−.07 (.07)	.80 [.24, 2.69]	2.70 [.33, 21.98]

5. Pronounced Negative Adol-Parent Divergence	−.45 (.36)	−.17 (.26)	.06 (.08)	−.02 (.12)	2.42 [.84, 6.99]	10.32* **[1.69, 63.11]**

4. *Negative Adol-Father Divergence*	*ref*	*ref*	*ref*	*ref*	*ref*	*ref*

5. Pronounced Negative Adol-Parent Divergence	−.41 (.39)	.18 (.28)	.06 (.08)	.04 (.12)	3.04 [.78, 11.91]	3.82 [.68, 21.48]

N Observations	504	506	514	515	522	515

Note:

* = *p* < *.05,*

** = *p* < *.01,*

*** = *p* < *.001,* estimates in bold text had p − values between .05 and .10 and 95% confidence intervals that did not contain 1. The four linear regression models adjusted for 6^th^ grade levels of outcomes. Logistic regression models for substance use initiation excluded those who had endorsed either having been drunk (n = 1) or using marijuana (n = 1) in 6^th^ grade. Redundant comparisons already shown in prior panels of the table were removed from subsequent panels. Adol. = Adolescent.
