# Supplementary material for: Examining profiles of convergence and divergence in reports of parental warmth: Links to adolescent developmental problems
Source: Dev Psychopathol. Author manuscript; Available in PMC 2025 Nov 1. (PMC11473715; doi:10.1017/S0954579424000762)
Supplement: 1 [file NIHMS1977836-supplement-1.docx]

**Supplemental Table 1**. *Comparison of Within-Profile Means*

|  | 1 | 2 | 3 | 4 | 5 |
| --- | --- | --- | --- | --- | --- |
|  | Positive Adolescent-Parent Divergence | Negative Adolescent-Mother Divergence | Negative Adolescent-Parent Divergence | Negative Adolescent-Father Divergence | Pronounced Negative Adolescent-Parent Divergence |
| *LPA Indicators* | (59%, n=404) | (22%, n=151) | (11%, n=74) | (5%, n=35) | (3%, n=20) |
| Comparisons to Profile 5 |  |  |  |  |  |
| Adol. Report of Father | 4.59*** | 3.59*** | 2.40*** | 1.56* | Ref |
| Adol. Report of Mother | 3.89*** | 2.61*** | 1.43*** | 3.48*** | Ref |
| Adol.-Father Discrepancy | 3.80*** | 3.21*** | 1.99*** | 1.78*** | Ref |
| Adol.-Mother Discrepancy | 3.05*** | 1.92*** | 0.96*** | 2.75*** | Ref |
| Comparisons to Profile 4 |  |  |  |  |  |
| Adol. Report of Father | 3.01*** | 2.01*** | 0.81*** | Ref |  |
| Adol. Report of Mother | 0.42*** | -0.86*** | -2.04*** | Ref |  |
| Adol.-Father Discrepancy | 1.02*** | 1.43*** | 0.22 | Ref |  |
| Adol.-Mother Discrepancy | 0.31*** | -0.83*** | -1.78*** | Ref |  |
| Comparisons to Profile 3 |  |  |  |  |  |
| Adol. Report of Father | 2.19*** | 1.19*** | Ref |  |  |
| Adol. Report of Mother | 2.46*** | 1.18*** | Ref |  |  |
| Adol.-Father Discrepancy | 1.78*** | 1.21*** | Ref |  |  |
| Adol.-Mother Discrepancy | 2.09*** | 0.96*** | Ref |  |  |
| Comparisons to Profile 2 |  |  |  |  |  |
| Adol. Report of Father | 0.99*** | Ref |  |  |  |
| Adol. Report of Mother | 1.28*** | Ref |  |  |  |
| Adol.-Father Discrepancy | 0.59*** | Ref |  |  |  |
| Adol.-Mother Discrepancy | 1.14*** | Ref |  |  |  |
| *Note:* Means were compared by running LPA models with indicator means centered around the means of the chosen reference profile. Indicator means are expressed here as deviations from the reference profile. Redundant comparisons are not shown. | | | | | |
