# Supplementary material for: Examining profiles of convergence and divergence in reports of parental warmth: Links to adolescent developmental problems
Source: Dev Psychopathol. Author manuscript; Available in PMC 2025 Nov 1. (PMC11473715; doi:10.1017/S0954579424000762)
Supplement: 2 [file NIHMS1977836-supplement-2.docx]

**Supplemental Table 2.** *Unadjusted associations between 6^th^ grade latent profiles and 9^th^ grade outcomes*

|  | Happiness | Life  Satisfaction* | Aggressive  Behavior* | Anxious /  Depressed | Been Drunk  Ever | Marijuana  Ever |
| --- | --- | --- | --- | --- | --- | --- |
|  | *Mean (SE)* | *Mean (SE)* | *Mean (SE)* | *Mean (SE)* | *Mean (SE)* | *Mean (SE)* |
| Sample Mean | 5.34 (1.16) | 4.78 (0.97) | 0.30 (0.28) | 0.26 (0.33) | 0.19 (0.39) | 0.09 (0.29) |
| *Latent Profile* |  |  |  |  |  |  |
| 1. Positive Adol.-Parent Divergence | 5.44 (0.07) | **4.94^2,4^ (0.05)** | **0.27^2,5^ (0.02)** | 0.23 (0.02) | 0.18 (0.02) | 0.09 (0.02) |
| 2. Negative Adol.-Mother Divergence | 5.14 (0.12) | **4.46^1^ (0.10)** | **0.37^1^ (0.03)** | 0.33 (0.04) | 0.20 (0.04) | 0.11 (0.03) |
| 3. Negative Adol.-Parent Divergence | 5.31 (0.19) | 4.70 (0.16) | 0.28 (0.04) | 0.28 (0.05) | 0.19 (0.06) | 0.03 (0.03) |
| 4. Negative Adol.-Father Divergence | 5.23 (0.22) | **4.45^1^ (0.20)** | 0.29 (0.05) | 0.24 (0.06) | 0.16 (0.09) | 0.07 (0.06) |
| 5. Pronounced Negative Adol.-Parent Divergence | 4.91 (0.34) | 4.54 (0.22) | **0.44^1^ (0.08)** | 0.32 (0.11) | 0.36 (0.12) | 0.24 (0.11) |
| *Note:* * Latent profile membership was an overall significant predictor of the outcome;  ^1^profile mean was significantly different from mean of Profile 1; ^2^profile mean was significantly different from mean of Profile 2; ^4^profile mean was significantly different from mean of Profile 4; ^5^profile mean was significantly different from mean of Profile 5  Statistical significance is at *p* < .05  Adol. = Adolescent | | | | | | |
|  |  |  |  |  |  |  |
